# Factors influencing mental health and oral health-related quality of life in population with recurrent aphthous stomatitis: a cross-sectional study

**DOI:** 10.3389/froh.2025.1652658

**Published:** 2025-10-29

**Authors:** Siwei Weng, Shujia Li, Sicong Hou

**Affiliations:** 1 Department of Stomatology, Yangzhou Hospital Affiliated to Nanjing University of Chinese Medicine, Yangzhou, Jiangsu, China; 2 Department of Stomatology, Clinical Traditional Chinese Medicine College of Yangzhou University, Yangzhou, Jiangsu, China; 3 Department of Dermatology, Yangzhou Hospital Affiliated to Nanjing University of Chinese Medicine, Yangzhou, Jiangsu, China; 4 The First School of Clinical Medicine, Faculty of Medicine, Yangzhou University, Yangzhou, China; 5 Key Laboratory of the Jiangsu Higher Education Institutions for Integrated Traditional Chinese and Western Medicine in Senile Diseases Control (Yangzhou University), Yangzhou, China

**Keywords:** recurrent aphthous stomatitis, dietary habits, TCM, mental health, OHRQoL

## Abstract

**Background:**

Recurrent episodes-induced oral functional limitations exacerbate anxiety, depression, and oral health-related quality of life (OHRQoL) impairments in Recurrent aphthous stomatitis (RAS) patients. However, the specific factors driving these psychological and OHRQoL deteriorations remain incompletely understood.

**Objectives:**

We aimed to quantify psychological distress and OHRQoL impairments in RAS patients and to identify modifiable risk factors underlying these deteriorations to inform targeted interventions.

**Methods:**

This cross-sectional study surveyed RAS patients via an online/paper questionnaire. Demographic data, clinical information, the level of anxiety, depression and OHRQoL were collected and analyzed.

**Results:**

Prolonged ulcer duration and preference for Traditional Chinese Medicine (TCM) interventions are significant risk factors for the development of anxious and depressive symptoms in RAS patients. Frequent consumption of fried foods (OR: 3.88, *p* = 0.006) increases the risk of anxiety. Patients with high fruit intake frequency (B: 3.42, *p* < 0.001) exhibit more severe anxiety symptoms. Spicy food intake (B: −1.18, *p* < 0.001) has a mitigating effect on anxiety. Anxious RAS patients with frequent vegetable intake (B: −4.820, *p* < 0.001) experience reduced anxiety levels. Larger ulcer diameter (B = 2.09, *p* = 0.017), higher ulcer recurrence frequency (B = 4.74, *p* < 0.001), and frequent consumption of fried foods (B = 2.19, *p* = 0.002) exacerbate depressive symptoms in depressive RAS patients. Worsening of pain, frequent consumption of fried foods (B = 2.68, *p* = 0.004), moderate fruit intake (B = 1.39, *p* = 0.019), and preference for TCM interventions (B = 2.08, *p* = 0.022) demonstrate poorer OHRQoL.

**Conclusions:**

Ulcer characteristics, dietary habits, and preference for TCM interventions were impairing risks for psychological distress and OHRQoL in RAS patients. Therefore, personalized psychological interventions should maintain control of mental health and OHRQoL, thereby reducing ulcer recurrence and improving outcomes.

## 1 Introduction

Recurrent Aphthous Stomatitis (RAS), one of the most prevalent oral mucosal disorders, is characterized by painful oral ulcers that recur over days to months, affecting approximately 25% of the global population (1). Although self-limiting, its recurrent nature significantly impairs patients' ability to masticate, speak, and swallow, thereby diminishing quality of life (2, 3). Current evidence indicates that RAS pathogenesis involves multifactorial interactions, such as immune dysregulation, genetic predisposition, psychological stress, hormonal fluctuations, local mechanical trauma, oral microbiome dysbiosis and diet (4, 5). Given the multifactorial etiology of RAS, its clinical management remains substantial challenging. Current therapeutic strategies mainly focus on symptomatic relief, including pain mitigation, ulcer healing acceleration, and recurrence prevention (6). Thus, elucidating the causative factors associated with RAS facilitates advances in comprehensive etiological understanding and enables the development of personalized prevention and treatment strategies tailored to individual etiological characteristics.

It is well known that oral chronic diseases are frequently associated with psychological comorbidities (7). Significantly elevated prevalence of depression and anxiety has been demonstrated in chronic oral conditions such as burning mouth syndrome (BMS) (8, 9) and periodontal disease (10). The bidirectional interaction between RAS and psychological status has been documented. Emotional stress has been identified as a triggering factor for acute RAS exacerbations (11). Conversely, RAS patients demonstrate significantly higher prevalence rates of anxiety, stress, and depression compared to healthy population. This phenomenon may stem from multi-factorial interactions involving oral functional impairments (e.g., pain during speech, mastication, and swallowing), impaired food/fluid intake, social interaction limitations, and compromised self-esteem, collectively contributing to deteriorations in both mental health (12). Consequently, attention to the psychological status of RAS patients is urgent and essential as an established contributor to RAS pathogenesis, dietary factors are frequently implicated (5). For example, a Chinese cross-sectional study identified a significant negative association between fruit consumption (an optimal source of antioxidants) and RAS occurrence (13). Additionally, adverse effects of spicy and fried foods (14), alongside specific food allergens (15), on RAS have been documented. A systematic review indicates that the “healthy” diet, characterized by a variety of vegetables, fruits, whole grains, and legumes, is associated with a reduced risk of depression or amelioration of depression symptoms (16). Similarly, diets rich in plant-based foods are linked to a decreased risk of anxiety (17). Nevertheless, studies exploring dietary impacts on psychological status in RAS population remain scarce. Integrating existing evidence, we incorporated dietary factors, specifically the intake frequency of vegetables, fruits, spicy foods, and fried foods, as variables influencing the psychological status of RAS patients. Furthermore. Traditional Chinese Medicine (TCM) has been utilized as a complementary and alternative medicine to Western medicine in China for several centuries, particularly in the chronic conditions (18). The chronic nature of RAS contributes to prolonged physical and psychological distress in patients, exacerbating levels of anxiety and depression (19). This amplifies the detrimental effects on oral health, thereby heightening the interest of clinicians and patients in alternative therapeutic approaches (20). The evidence indicates that topical application of natural herbal medicines demonstrates efficacy in improving the prognosis of RAS while mitigating adverse effects (34). Since a proportion of patients in TCM hospitals exhibit an intention to choose TCM treatment, we included preference for TCM as an influencing factor.

The assessment of health-related quality of life (HRQoL) holds significant clinical value in healthcare and has been established as a widely accepted primary endpoint in clinical research trials (21). Given the profound impact of oral diseases on systemic health and overall well-being, oral health-related quality of life (OHRQoL) evaluation has become an integral component of oral disease management (22). Existing evidence highlights. Furthermore, patients with oral lichen planus typically exhibit poorer OHRQoL compared to healthy controls, with pharmacological interventions yielding measurable improvements in their OHRQoL (21). A small-scale prospective study (*N* = 62) revealed that individuals with RAS experience substantially more pronounced adverse effects on OHRQoL than both healthy controls and those in ulcer remission phases (23). Despite existing evidence confirming poorer psychological status and significantly worse OHRQoL in RAS patients compared to healthy controls, the specific contributing factors underlying these abnormalities remain incompletely understood. Therefore, this study aims to: 1. Quantify the severity of psychological disturbances and OHRQoL levels in RAS patients; 2. Identify key factors influencing their psychological status and OHRQoL; 3. Conduct the first systematic investigation into factors modulating the severity of psychological comorbidities, thereby informing the development of targeted intervention strategies to optimize clinical outcomes and patient-centered therapy strategies.

This study demonstrated the multifactorial influnce of dietary behaviors, ulcer characteristics, pain intensity, and propensity for TCM intervention on psychological status and OHRQoL in patients with RAS. Furthermore, it delineated the differential impacts of these variables on anxiety/depression severity within RAS population. Our data provides evidence to understand the psychological status and OHRQoL of RAS population. The findings inform the development of tailored interventions to mitigate RAS-related impairments in OHRQoL and mental health. The results seek to address the distinct clinical challenges in this population, contributing to the development of therapeutic strategies that may ultimately improve treatment efficacy and quality of life.

## 2 Methods

### 2.1 Participants

This cross-sectional study was conducted from March 2024 to March 2025. The cohort exclusively recruited adults aged ≥18 years, with exclusion criteria encompassing psychiatric conditions, cognitive dysfunction, communication disabilities, or illiteracy. A definitive RAS diagnosis was confirmed through a protocol requiring complete fulfillment of all major diagnostic criteria and at least one minor criterion, based on validated RAS assessment tools integrating both symptomatic manifestations and physical examination findings (24).

The major diagnostic criteria encompass: (i) Morphological features-multiple small oval-shaped recurrent ulcers with well-defined erythematous halos and yellow or gray bases; (ii) Recurrence pattern-average annual frequency ≥1 episode without site-specific predilection; (iii) Mechanical allodynia-pain symptoms exacerbated by tactile stimuli; (iv) Self-limiting nature-spontaneous healing without therapeutic intervention. Minor criteria include: (i) Positive family history-at least one first-degree relative with confirmed RAS; (ii) Anatomical localization-involvement of non-keratinized oral mucosa; (iii) Duration-persistence from several days to two weeks; (iv) Triggering factors-stress, local trauma, or infection; (v) Non-smoker status. Furthermore, we excluded subjects presenting with localized oral traumatic factors or systemic comorbidities (including hematological deficiencies, Crohn's disease, Behçet's syndrome, autoinflammatory disorders, endocrine/metabolic dysregulations such as diabetes, as well as hypertension, cardiovascular, renal, gastrointestinal, or hepatic pathologies).

### 2.2 Data collection

OHRQoL, anxiety, and depression were assessed via online or paper-based questionnaires. A total of 400 questionnaires (online/paper) were randomly distributed, with 42 subjects excluded from the final analysis: 12 were diagnosed with psychiatric disorders during the study period, 24 presented psychosomatic comorbidities strongly associated with psychological status, and 6 were removed due to insufficient data completeness. The sample size met the statistical power requirements for subsequent analyses, based on methodological frameworks proposed by Peduzzi et al. (25) and Concato et al. (26). All protocols adhered strictly to the ethical principles of the Declaration of Helsinki and were pre-approved by the Institutional Review Board and Ethics Committee of Clinical Chinese Medicine at Yangzhou University (REC ref 2024-54). Written informed consent was obtained from all participants.

The first section of the questionnaire collected participants' basic demographic information, including gender, age, occupation, educational level, income, and marital status. The second section included RAS-related questions, such as: What is the diameter of the largest ulcer in the oral cavity? Is the frequency of annual episodes below 7 or above 7? Does the duration of ulcer episodes exceed 14 days or remain under 14 days? Was seeking treatment at this institution motivated by a request for TCM interventions? On a Numerical Rating Scale (NRS), how would you rate the pain intensity during ulcer episodes? The NRS is a concise clinical instrument based on self-reported symptom intensity, widely utilized for systematic quantitative evaluation of pain severity. This scale requires participants to assign a numerical score (0–10) to specific symptoms or clinical issues based on their subjective experience, thereby transforming subjective complaints into quantifiable objective data to aid clinicians in precisely interpreting patients' pain perception profiles. The NRS employs a 10-point grading system: 0 indicates no pain or symptoms; 1–3 corresponds to mild pain (no significant interference with daily activities or sleep); 4–6 suggests moderate pain (with compromised sleep quality); and 7–10 denotes severe pain (marked sleep disruption accompanied by substantial physical and psychological burden) (27).

The third section focused on evaluating dietary patterns in patients with RAS. Following the framework of the Dietary Guidelines for Chinese Residents (28) issued by the Chinese Nutrition Society, we investigated the consumption frequency of key dietary factors, including fruits, vegetables, spicy foods, and fried foods. Consumption frequency was categorized into three standardized tiers: regular intake (defined as ≥4 times/week), occasional intake (1–3 times/week), and no/rare intake.

The fourth section focused on assessing respondents' psychological status and quality of life. The Generalized Anxiety Disorder-7 (GAD-7) scale was used to evaluate the severity of anxiety over the past month. This scale consists of 7 items, each scored on a 0–3 scale (0 = not at all; 3 = nearly every day), with a total score ranging from 0 to 21. Higher total scores indicate greater anxiety severity. A score of 0–4 was defined as no clinically significant anxiety, while a score ≥5 indicated clinically significant anxiety (29).

The Patient Health Questionnaire-9 (PHQ-9) was used to assess depressive status. This scale consists of 9 items, each scored 0–3, yielding a total score range of 0–27. A score of 0–4 is defined as normal status, while a score ≥5 indicates clinically significant depression, with higher total scores reflecting greater depression severity (30). The Oral Health Impact Profile-14 (OHIP-14) was employed to evaluate participants' OHRQoL. This instrument covers 14 items across 7 domains, each offering 5 response options. Individual item scores range from 0 to 4, with a total score of 0–56 inversely correlated with OHRQoL levels (31).

### 2.3 Statistical analysis

Statistical analyses were performed using SPSS version 27.0 (IBM SPSS Statistics, USA). Continuous variables are expressed as mean ± standard deviation (SD), while categorical variables are presented as proportions. Univariate logistic regression analysis was used to identify potential moderators of psychological stress occurrence. Candidate predictors were screened with a threshold of *p* < 0.05 in univariate analysis. Subsequently, variables without statistical significance were excluded via multivariate analysis. A *p* < 0.05 in multivariate analysis was considered statistically significant. The association between variables and psychological stress outcomes was measured using odds ratios (OR). Multiple linear regression analyses were performed using OHIP-14 scores of RAS patients and GAD-7 or PHQ-9 scores of RAS patients with clinically significant anxiety or depression as continuous outcomes. Beta coefficients and 95% confidence intervals for *p*-values were calculated.

## 3 Results

A total of 400 online questionnaires were distributed among the RAS population, with 42 excluded from analysis. Baseline characteristics are summarized in Table 1. The majority of participants were aged 35–64, with a male-to-female ratio of 148:210. Married individuals accounted for 38.55%, while 61.45% were single. Occupationally, 27.65% worked in state-owned enterprises, 27.09% in private enterprises, 26.26% were self-employed, and 18.99% were retired. Regarding educational attainment, 46.65% held a bachelor's degree. Smokers and alcohol consumers comprised 18.16% and 14.80%, respectively. For RAS-related parameters, only 3.63% of patients reported no pain symptoms, while 80 exhibited severe pain (NRS scores ≥7). A total of 106 patients (29.61%) reported ulcer diameters >1 cm. Patients with ulcer recurrence frequency ≥7 episodes/year accounted for 55.03%, while 72 patients exhibited episode duration ≥14 days. Over half of RAS patients sought TCM-based interventions at our institution (66.20%). Regarding dietary patterns, 213 participants (59.49%) consumed fried foods 1–3 times/week, whereas only 55 (15.36%) never consumed spicy foods, 43 (12.01%) never consumed vegetables, and 86 (18.16%) consumed fruits ≥4 times/week. Using a cutoff score of 5 on the GAD-7 scale, the anxiety prevalence rate was 49.16% (176/358), distributed as 128 mild, 30 moderate, and 18 severe cases (Table 2). For depression (PHQ-9 cutoff ≥5), 103 patients (28.77%) met criteria for depression, including 67 mild, 19 moderate, and 17 severe cases. The mean OHIP-14 score for OHRQoL in RAS patients was 21.06 ± 8.39.

**Table 1:** Demographic characteristics of patients included in the study.

Variables	*n* (%)
Gender	
Male	148 (41.34%)
Female	210 (58.66%)
Age, years	
18–34	100 (27.93%)
35–64	139 (38.83%)
65+	119 (33.24%)
Marital status	
Married	138 (38.55%)
Single	220 (61.45%)
Occupation	
State-owned units	99 (27.65%)
Private units	97 (27.09%)
Freelance work	94 (26.26%)
Retired	68 (18.99%)
Education level	
Middle school	115 (32.12%)
Bachelor	167 (46.65%)
Above Bachelor	76 (21.23%)
Socio-economic status	
Low	90 (25.14%)
Middle	104 (29.05%)
High	164 (45.81%)
Smoking	65 (18.16%)
Drinking	53 (14.80%)
Numerical rating scale (0–10)	
NRS = 0 (no pain)	13 (3.63%)
NRS = 1–3 (mild pain)	126 (35.19%)
NRS = 4–6 (moderate pain)	139 (38.83%)
NRS = 7–10 (severe pain)	80 (22.35%)
Ulcer diameter	
≤1 cm	252 (70.39%)
>1 cm	106 (29.61%)
Seizure frequency	
<7 episodes/year	161 (44.97%)
≥7 episodes/year	197 (55.03%)
Duration	
<14 days	286 (79.89%)
≥14 days	72 (20.11%)
Preference for TCM interventions	237 (66.20%)
Fried foods	
Never or seldom	71 (19.83%)
Sometimes	213 (59.49%)
Often	74 (20.67%)
Spicy foods	
Never or seldom	55 (15.36%)
Sometimes	210 (58.66%)
Often	93 (25.98%)
Vegetables	
Never or seldom	43 (12.01%)
Sometimes	221 (61.73%)
Often	94 (26.26%)
Fruit	
Never or seldom	103 (34.92%)
Sometimes	169 (46.93%)
Often	86 (18.16%)

**Table 2:** Level of anxiety, depression and OHRQoL among patients included in the study.

Variables	*n* (%)	Mean ± SD
Anxiety (GAD-7)		
No	182 (50.84%)	1.91 ± 1.13
Mild	128	6.84 ± 1.26
Moderate	30	11.73 ± 1.31
Moderately severe	18	17.33 ± 1.64
Depression (PHQ-9)		
No	255 (71.23%)	2.11 ± 0.84
Mild	67	6.97 ± 1.25
Moderate	19	11.79 ± 1.32
Moderately severe	17	16.35 ± 1.22
Severe	0	
OHRQoL (OHIP-14)		21.06 ± 8.39

To evaluate the impact of these variables on anxiety and depression occurrence, we first screened potential factors using univariate logistic regression (Tables 3, 4). Multivariate analysis revealed that prolonged duration of ulcer episodes (OR: 11.95, *p* < 0.001) and preference for TCM interventions (OR: 13.20, *p* < 0.001) were independent risk factors for anxiety. High-frequency consumption of fried foods (OR: 3.88, *p* = 0.006) and relatively moderate fruit intake (OR: 3.70, *p* < 0.001) also increased anxiety risk, whereas ulcer diameter >1 cm (OR: 0.29, *p* = 0.004) reduced anxiety likelihood. For depression, age 35–64 years (OR: 2.38, *p* = 0.049), 65 + years (OR: 2.74, *p* = 0.029), ulcer duration ≥2 weeks (OR: 5.14, *p* < 0.001), preference for TCM interventions (OR: 6.51, *p* = 0.005), fried food consumption 1–3 times/week (OR: 13.20, *p* < 0.001)and ≥3 times/week,(OR: 13.20, *p* < 0.001) and vegetable intake ≥4 times/week (OR: 19.53, *p* < 0.001)were identified as risk factors. The significantly elevated depression risk in the 35–64 (OR: 2.38, *p* = 0.049) and 65+ (OR: 2.74, *p* = 0.029) age groups may reflect the dual burden of sociocultural stressors (e.g., familial responsibilities, occupational competition) and age-related physiological decline in middle-aged and elderly population (32).

**Table 3:** Factors of the occurrence of anxiety.

Variables	Univariable analysis		Multivariable analysis			
	*p*	OR	95% Cl	*p*	OR	95% Cl
Gender (male)	<0.001*	2.237	1.456–3.438	0.291	1.361	0.768–2.413
Age, year						
18–34						
35–64	0.529	0.847	0.506–1.419			
65+	0.220	1.396	0.818–2.381			
Marital status (single)	0.182	1.338	0.873–2.050			
Occupation						
State-owned units						
Private units	0.872	0.955	0.543–1.678			
Freelance work	0.051	1.763	0.996–3.119			
Retired	0.372	1.326	0.714–2.462			
Education level						
Middle school						
Bachelor	0.742	0.909	0.517–1.600			
Above Bachelor	0.247	0.737	0.440–1.235			
Socio-economic status						
Low						
Middle	0.0720	1.555	0.961–2.515			
High	<0.001*	2.177	1.207–3.929	0.167	1.708	0.799–3.652
Smoking	0.029*	1.847	1.066–3.201	0.091	1.859	0.905–3.822
Drinking	0.134	0.635	0.350–1.151			
Ulcer diameter (>1 cm)	<0.001*	2.254	1.413–3.595	0.004*	0.295	0.130–0.670
Seizure frequency (≥7 episodes/year)	<0.001*	3.387	2.187–5.245	0.714	1.154	0.537–2.479
Duration (≥14 days)	<0.001*	7.328	3.775–14.223	<0.001*	11.946	5.069–28.151
Preference for TCM interventions	<0.001*	6.300	3.791–10.469	<0.001*	13.201	4.847–35.956
Numerical rating scale (0–10)						
NRS = 0 (no pain)						
NRS = 1–3 (mild pain)	0.055	4.543	0.967–21.341			
NRS = 4–6 (moderate pain)	0.013*	7.033	1.503–32.918	0.274	3.704	0.354–38.736
NRS = 7–10 (severe pain)	0.039*	5.232	1.089–25.125	0.769	1.429	0.133–15.383
Fried foods						
Never or seldom						
Sometimes	<0.001*	2.623	1.465–4.697	0.123	1.900	0.841–4.296
Often	<0.001*	4.708	2.329–9.515	0.006*	3.877	1.489–10.100
Spicy foods						
Never or seldom						
Sometimes	0.995	0.998	0.551–1.808			
Often	0.965	1.015	0.521–1.977			
Vegetables						
Never or seldom						
Sometimes	0.250	0.681	0.354–1.311			
Often	0.101	1.849	0.887–3.855			
Fruit						
Never or seldom						
Sometimes	0.002*	2.219	1.353–3.639	<0.001*	3.703	1.984–6.911
Often	0.142	1.543	0.864–2.754			

* *p* < 0.05 was considered significant.

**Table 4:** Factors of the occurrence of depression.

Variables	Univariable analysis		Multivariable analysis			
	*p*	OR	95% Cl	*p*	OR	95% Cl
Gender (male)	0.295279	1.280	0.806–2.031			
Age, year						
18–34						
35–64	0.030*	1.975	1.068–3.650	0.049*	2.382	1.006–5.642
65+	0.016*	2.159	1.152–4.044	0.029*	2.740	1.106–6.788
Marital status (single)	0.375	0.806	0.501–1.298			
Occupation						
State-owned units						
Private units	0.805	1.084	0.573–2.052			
Freelance work	0.306	1.388	0.741–2.598			
Retired	0.317	1.416	0.717–2.797			
Socio-economic status						
Low						
Middle	0.574	0.842	0.464–1.531			
High	0.036*	0.547	0.311–0.961	0.814	0.907	0.403–2.041
Education level						
Middle school						
Bachelor	0.662	1.125	0.662–1.911			
Above Bachelor	0.620	1.176	0.620–2.229			
Smoking	0.464	1.228	0.688–2.191			
Drinking	0.935	0.973	0.510–1.859			
Ulcer diameter (>1 cm)	<0.001*	5.934	3.590–9.809	0.994	0.996	0.362–2.744
Seizure frequency (≥7 episodes/year)	<0.001*	6.029	3.425–10.613	0.215	1.763	0.720–4.317
Duration (≥14 days)	<0.001*	8.400	4.743–14.875	<0.001*	5.136	1.960–13.458
Preference for TCM interventions	<0.001*	4.596	2.482–8.509	0.005*	6.515	1.749–24.265
Numerical rating scale (0–10)						
NRS = 0 (no pain)						
NRS = 1–3 (mild pain)	0.807	1.183	0.307–4.562			
NRS = 4–6 (moderate pain)	0.239	0.434	0.108–1.743			
NRS = 7–10 (severe pain)	0.011*	5.862	1.492–23.032	0.491	2.355	0.206–26.881
Fried foods						
Never or seldom						
Sometimes	0.006*	2.893	1.355–6.178	0.244	2.016	0.619–6.564
Often	<0.001*	4.966	2.149–11.480	0.683	0.770	0.219–2.702
Spicy foods						
Never or seldom						
Sometimes	0.509	1.262	0.633–2.519			
Often	0.214	1.615	0.758–3.443			
Vegetables						
Never or seldom						
Sometimes	0.336	1.629	0.602–4.403			
Often	<0.001*	12.811	4.611–35.596	<0.001*	19.530	5.359–71.179
Fruit						
Never or seldom						
Sometimes	0.643	1.135	0.665–1.939			
Often	0.977	1.009	0.533–1.912			

* *p* < 0.05 was considered significant.

To explore factors influencing anxiety severity among anxious individuals, we screened potential variables using univariate logistic regression in RAS patients with GAD-7 scores ≥5 (Table 5). Significant variables primarily related to dietary habits. Compared to rare/never fruit consumption or intake 1–3 times/week, frequent fruit consumption (≥4 times/week) (B: 3.42, *p* < 0.001) was associated with more severe anxiety symptoms. However, regular vegetable consumption was linked to reduced anxiety severity compared to minimal vegetable intake, and moderate spicy food intake (B: −1.18, *p* = 0.004) also correlated with lower anxiety levels in this population.

**Table 5:** Factors associated with elevated anxiety.

Variables	Univariable analysis		Multivariable analysis			
	*p*	B	95% Cl	*p*	B	95% Cl
*p*	B	95% Cl	*p*	B	95% Cl
Gender (male)	0.347	0.523	−0.571–1.617			
Age, year						
18–34						
35–64	0.106	−1.145	−2.538–0.248			
65+	0.080	−1.216	−2.581–0.149			
Marital status (single)	0.280	−0.608	−1.715–0.499			
Occupation						
State-owned units						
Private units	0.928	0.070	−1.473–1.614			
Freelance work	0.083	−1.277	−2.724–0.170			
Retired	0.033*	−1.763	−3.383–(−0.143)	0.935	0.028	−0.661–0.718
Socio-economic status						
Low						
Middle	0.268	0.813	−0.630–2.256			
High	0.772	−0.197	−1.536–1.142			
Education level						
Middle school						
Bachelor	0.031*	−1.437	−2.742–(−0.133)	0.948	0.023	−0.702–0.657
Above Bachelor	0.006*	−2.112	−3.606–(−0.617)	0.593	−0.212	−0.993–0.569
Smoking	0.725	0.234	−1.074–1.542			
Drinking	0.513	0.561	−1.129–2.250			
Ulcer diameter (>1 cm)	0.315	−0.575	−1.701–0.551			
Seizure frequency (≥7 episodes/year)	<0.001*	2.577	1.446–3.708	0.732	0.117	−0.557–0.792
Duration (≥14 days)	0.185	−0.775	−1.926–0.376			
Preference for TCM interventions	0.005*	2.182	0.672–3.692	0.558	0.320	−0.757–1.398
Numerical rating scale (0–10)						
NRS = 0 (no pain)						
NRS = 1–3 (mild pain)	0.809	0.553	−3.9620–5.068			
NRS = 4–6 (moderate pain)	0.415	1.859	−2.635–6.353			
NRS = 7–10 (severe pain)	0.016*	5.603	1.052–10.153	0.267	0.542	−0.420–1.504
Fried foods						
Never or seldom						
Sometimes	0.373	0.685	−0.829–2.199			
Often	<0.001*	4.854	3.199–6.509	0.650	−0.196	−1.046–0.655
Spicy foods						
Never or seldom						
Sometimes	<0.001*	−4.017	−5.250–(−2.784)	0.004*	−1.181	−1.971–(−0.391)
Often	0.186	0.931	−0.451–2.313			
Vegetables						
Never or seldom						
Sometimes	<0.001*	−7.958	−8.992–(−6.923)	<0.001*	−4.174	−5.579–(−2.767)
Often	<0.001*	−9.563	−10.644–(−8.482)	<0.001*	−4.820	−6.444–(−3.195)
Fruit						
Never or seldom						
Sometimes	0.065	0.683	−0.043–1.409			
Often	<0.001*	7.464	6.612–8.316	<0.001*	3.418	2.273–4.563

* *p* < 0.05 was considered significant.

In the RAS patient population with PHQ-9 scores ≥5 (indicating clinically significant depression), multivariate analysis was conducted to explore factors influencing depression severity after screening potential variables via univariate logistic regression (Table 6). Ulcer diameter >1 cm (B = 2. 09, *p* = 0.02), annual recurrence frequency ≥7 episodes (B = 4.74, *p* < 0.001), and high-frequency fried food consumption (B = 2.19, *p* = 0.002) were identified as independent factors contributing to depression progression. Conversely, a preference for TCM interventions (B = −3.70, *p* = 0.023) and high-frequency vegetable consumption (B = −3.33, *p* < 0.001) were associated with reduced depression severity in this cohort.

**Table 6:** Factors associated with elevated depression.

Variables	Univariable analysis			Multivariable analysis		
	*p*	B	95% Cl	*p*	B	95% Cl
Gender (male)	0.068	−1.376	−2.856–0.104			
Age, year						
18–34						
35–64	0.894	0.141	−1.945–2.228			
65+	0.495	0.141	−2.849–1.386			
Marital status (single)	0.654	0.355	−1.215–1.926			
Occupation						
State-owned units						
Private units	0.491	−0.740	−2.864–1.384			
Freelance work	0.590	0.560	−1.494–2.614			
Retired	0.424	0.896	−1.321–3.113			
Socio-economic status						
Low						
Middle	0.270	−1.042	−2.905–0.822			
High	0.062	−1.717	−3.519–0.085			
Education level						
Middle school						
Bachelor	0.390	0.741	−0.960–2.441			
Above Bachelor	0.014*	2.560	0.520–4.599	0.167	1.003	−0.426–2.432
Smoking	0.291	−0.991	−2.841–0.859			
Drinking	0.949	0.069	−2.056–2.194			
Ulcer diameter (>1 cm)	0.001*	2.378	0.938–3.819	0.017*	2.092	0.377–3.806
Seizure frequency (≥7 episodes/year)	0.003*	2.918	1.030–4.805	<0.001*	4.743	2.386–7.100
Duration (≥14 days)	0.031*	1.616	0.148–3.084	0.190	1.056	−0.533–2.645
preference for TCM interventions	0.010*	2.787	0.669–4.903	0.0232*	−3.703	−6.876–(−0.529)
Numerical rating scale (0–10)						
NRS = 0 (no pain)						
NRS = 1–3 (mild pain)	0.596	−1.121	−5.302–3.060			
NRS = 4–6 (moderate pain)	0.266	−2.458	−6.821–1.904			
NRS = 7–10 (severe pain)	0.429	1.647	−2.472–5.766			
Fried foods						
Never or seldom						
Sometimes	0.599	0.683	−1.889–3.255			
Often	0.019*	3.290	0.557–6.024	0.002*	2.186	0.796–3.576
Spicy foods						
Never or seldom						
Sometimes	0.246	0.282	−1.993–2.556			
Often	0.163	−1.737	−4.189–0.716			
Vegetables						
Never or seldom						
Sometimes	0.657	0.495	−1.711–2.699			
Often	0.023*	−2.489	−4.629–(−0.348)	<0.001*	−3.335	−4.723–(−1.946)
Fruit						
Never or seldom						
Sometimes	0.353	−0.827	−2.584–0.931			
Often	0.809	−0.258	−2.367–1.851			

* *p* < 0.05 was considered significant.

Prolonged ulcer duration (B = 3.44, *p* < 0.001), preference for TCM interventions (B = 2.08, *p* = 0.022), higher self-rated pain scores, frequent fried food consumption (B = 2.68, *p* = 0.004), and moderate fruit intake (1–3 times/week) (B = 1.39, *p* = 0.019) were identified as independent risk factors for impaired OHRQoL (Table 7). We summarize the impacts of various factors on anxiety, depression, and OHRQoL in Table 8.

**Table 7:** Factors associated with OHRQoL.

Variables	B	Std. error	Beta	*t*	*p*	95% CI for B
Gender (male)	0.880141	0.550946	0.051709	1.597508	0.111112	−0.203681
Marital status (single)	0.295392	0.499336	0.017152	0.591570	0.554545	−0.686902
Age, year						
18–34						
35–64	−0.926488	0.604860	−0.053870	−1.531739	0.126548	−2.116370
65+	0.624415	0.667101	0.035093	0.936013	0.349953	−0.687906
Occupation						
State-owned units						
Private units	−0.880721	0.663	−0.043	−1.213	0.226	−2.108
Freelance work	0.070763	0.663	0.012	0.347	0.729	−1.075
Retired	−1.145711	0.783	−0.053617	−1.462562	0.144542	−2.686736
Socio-economic status						
Low						
Middle	0.254468	0.675586	0.013783	0.376663	0.706667	−1.074545
High	0.093926	0.627	0.005583	0.149611	0.881163	−1.141085
Education level						
Middle school						
Bachelor	−0.176121	0.563971	−0.010482	−0.312288	0.755019	−1.285565
Above Bachelor	0.024340	0.689890	0.001187	0.035281	0.971877	−1.332812
Smoking	0.326573	0.639565	0.015019	0.510617	0.609961	−0.931580
Drinking	−0.442068	0.698903	−0.018731	−0.632516	0.527489	−1.816950
Ulcer diameter (>1 cm)	−1.286967	0.855645	−0.070096	−1.504091	0.133517	−2.970193
Seizure frequency (≥7 episodes/year)	0.359289	0.796122	0.021324	0.451298	0.652072	−1.206844
Duration (≥14 days)	3.442	0.878	0.165	3.922	<0.001*	1.716
Preference for TCM interventions	2.077	0.904	0.117	2.296	0.022*	0.298
Numerical Rating Scale (0–10)						
NRS = 0 (no pain)						
NRS = 1–3 (mild pain)	4.505459	1.395743	0.256710	3.227999	0.001372*	1.759751
NRS = 4–6 (moderate pain)	12.376157	1.495251	0.719597	8.276976	<0.001*	9.434698
NRS = 7–10 (severe pain)	18.901399	1.525620	0.939368	12.389319	<0.001*	15.900197
Fried foods						
Never or seldom						
Sometimes	1.447813	0.793901	0.084793	1.823669	0.069109	−0.113950
Often	2.684344	0.912800	0.129684	2.940779	0.003506*	0.888683
Spicy foods						
Never or seldom						
Sometimes	−1.394341	0.842936	−0.081919	−1.654148	0.099051	−3.052566
Often	−0.449115	0.921043	−0.023496	−0.487615	0.626147	−2.260991
Vegetables						
Never or seldom						
Sometimes	−1.132621	0.922951	−0.065677	−1.227174	0.220635	−2.948251
Often	−1.884366	0.998163	−0.098925	−1.887833	0.059928	−3.847953
Fruit						
Never or seldom						
Sometimes	1.388838	0.587050	0.082629	2.365790	0.018571*	0.233992
Often	1.360459	0.731759	0.068496	1.859164	0.063897	−0.079057

* *p* < 0.05 was considered significant.

**Table 8:** The impact of various factors on anxiety, depression, and OHRQoL.

Variables	The occurrence of anxiety	The occurrence of depression	Elevated anxiety	Elevated depression	OHRQoL
Age, year					
35–64		△			
65+		△			
Ulcer diameter (>1 cm)	○			△	
Seizure frequency (≥7 episodes/year)				△	
Duration (≥14 days)	△	△			△
Preference for TCM interventions	△	△		○	△
Numerical rating scale (0–10)					
NRS = 1–3 (mild pain)					△
NRS = 4–6 (moderate pain)					△
NRS = 7–10 (severe pain)					△
Fried foods (Often)	△			△	△
Spicy foods ( Sometimes)			○		
Vegetables					
Sometimes			○		
Often		△	○	○	
Fruit					
Sometimes	△				△
Often			△		

△, the symbol denotes a risk factor; ○, the symbol denotes a protective factor.

## 4 Discussion

This cross-sectional study systematically investigates the manifestations of anxiety and depression and the impairments of OHRQoL in RAS population. Through standardized patient-reported questionnaires. By innovatively incorporating dietary patterns, ulcer characteristic, pain severity, and propensity for TCM interventions as core covariates, we elucidated the multivariate associations between these predictors and psychological distress (anxiety/depression) alongside OHRQoL.

This study identified prolonged ulcer duration and TCM-seeking propensity as shared risk factors significantly contributing to the occurrence of anxiety and depressive symptoms in RAS patients. In patients with chronic oral conditions such as BMS, even those without overt burning pain symptoms demonstrated comparable rates of anxiety and depressive comorbidities mediated by BMS-related orofacial sensorimotor dysfunction (e.g., dysgeusia or masticatory dystonia), which were statistically equivalent to those observed in the BMS subgroup with characteristic burning sensations (33).Our findings suggest that prolonged ulcer duration elevates psychological distress risk through mediating pathways involving cumulative burdens of prolonged oral functional impairments (e.g., speech/swallowing deficits lasting >14 days). Notably, the study cohort was recruited from a TCM hospital, thus including individuals with a behavior characterized by TCM preference. In our study, preference for TCM was significantly associated with elevated risks of anxiety and depression in RAS patients. This is the first investigation addressing the impact of TCM preference to mental status in RAS population. A nationwide questionnaire survey across 31 provincial capitals in China (*N* = 31,599 outpatients completing all treatment procedures) revealed that this cohort generally perceived TCM as effective in all stages of holistic disease treatment for complex diseases, particularly in disease prevention, pain alleviation, and quality-of-life enhancement. Moreover, individuals from TCM hospitals demonstrated significantly more positive attitudes toward TCM compared to those from other medical institutions (34). A discrete choice experiment conducted across six provinces in China (*N* = 2,019 residents) revealed that respondents showed a stronger preference for TCM services in terms of treatment attributes under hypothetical scenarios of severe chronic diseases (35). We posit that RAS patients seek TCM due to pessimistic expectations toward conventional therapies and negative perceptions of functional impairment caused by RAS. These individuals may experience greater psychological burden, the similar phenomenon observed in other chronic conditions with suboptimal therapeutic outcomes, exemplified by patients with refractory atrial fibrillation who frequently develop heightened anxiety and depression (36), owing to the debilitating impact of symptoms, disillusionment from repeated treatment failures, and the unpredictability of symptom onset (37).However, among RAS population with depressive symptoms, TCM preference mitigated depression severity. This phenomenon is probably mediated by positive expectations regarding TCM efficacy within the RAS population, as the evidence indicates that optimistic treatment anticipation can alleviate emotional distress (38).This phenomenon is well-documented in clinical practice, wherein positive treatment expectations can indeed yield therapeutic benefits (39).

Within RAS characteristics, this study found that larger ulcer diameter exacerbated depressive symptoms in RAS patients with depression. Patients with chronic diseases often endure significant mental health burdens due to prolonged disease courses, recurrent episodes, and resultant constraints on lifestyle and functional capacity (40). For instance, depression severity correlates strongly with chronic diseases such as diabetes, pulmonary disorders, cardiac conditions, and arthritis (41). Particularly, patients with noncommunicable chronic diseases exhibit more severe depression than those with communicable chronic diseases (42). Larger ulcer diametersimpair oral function and disrupt daily life routines in RAS patients, thereby inducing more severe depression. In our study, elevated recurrence frequency exacerbated depressive severity, aligning with prior research. This correlation stems from the chronicity of the disease, persistent symptoms, recurrent episodes, and suboptimal therapeutic outcomes, which collectively engender patients' perception of an inability to alter or control their condition. This perception further contributes to negative emotions and psychological distress (43). This pattern of recurrent symptoms triggering negative emotions is also observed in other chronic and relapsing conditions, such as chronic obstructive pulmonary disease and systemic lupus erythematosus (44). Interestingly, ulcer diameter emerged as a protective factor against anxiety development, potentially mediated by increased medical help-seeking behaviors triggered by larger ulcer size. This observation aligns with problem-focused coping mechanisms, which are epidemiologically associated with reduced psychological morbidity in chronic disease population (45).

Notably, this study further elucidated the differential impacts of dietary patterns on psychoemotional comorbidities. High-frequency fried food consumption was observed to significantly increase anxiety risks and exacerbation of depressive severity. A nationwide population-based cohort study (*n* = 140,728) demonstrated that frequent consumption of fried potato products was significantly associated with a 12% increased risk of anxiety disorders [hazard ratio (HR): 1.12; 95% CI: 1.06–1.18; *p* < 0.001 for trend] and a 7% elevated incidence of depressive symptoms (HR = 1.07, 95% CI: 1.02–1.12, *p* < 0.001 for trend) (46). Based on this evidence, it is recommended that RAS patients, particularly those with comorbid anxiety-depression, should restrict fried food intake as a key dietary management objective. Our study identified a positive association between increased fruit intake frequency and anxiety incidence in RAS patients, alongside exacerbated anxiety severity among those with pre-existing anxiety. This finding appears inconsistent with prior research, such as the HEI-2020 study linking high-quality diets (rich in fruits/vegetables and low in added sugars) to reduced anxiety risk (47). Notably, 52.6% of Chinese participants intentionally increased fruit consumption during ulcer episodes, likely due to beliefs that fruit-derived vitamins aid ulcer healing and hydration (48). A plausible explanation is that frequent snacking on fruits during active RAS phases may exacerbate oral discomfort through mechanical irritation during mastication and swallowing, thereby triggering psychological distress. Moreover, fruit varieties are also implicated in the occurrence and progression of RAS. Studies indicate that certain fruits, such as oranges, lemons, and pineapples, can induce pro-inflammatory cascades and exacerbate RAS symptoms (49).We postulate that the resulting exacerbation of RAS symptoms may thereby induce psychological distress. The study also revealed that spicy food intake may exert potential anxiety-alleviating effects. Notably, spicy components are prevalent across global dietary cultures; for instance, in China, over 30% of adults consume spicy foods daily (50). Furthermore, a study on taste-emotion associations demonstrated that spicy taste stimuli ranked second only to sweetness in their linkage to positive emotional terms (51), and this association may partially explain its mechanistic role as a protective factor against anxiety level. In this study, higher-frequency vegetable intake reduced depression severity in RAS patients with depressive symptoms and alleviated anxiety levels in those with anxiety disorders. This aligns with prior research demonstrating that a 12-week intervention increasing vegetable consumption effectively mitigates depressive symptoms, thereby exerting therapeutic effects on clinically depressed population (13). Additional evidence indicates that vegetable intake correlates with reduced depression and anxiety symptoms, while dietary patterns rich in vegetables and omega-3 fatty acids are associated with improved mental health status, positively influencing emotional well-being (52). Interestingly, high vegetable frequency increased depression risk in our RAS cohort. We posit that mechanical friction from that increased frequency of vegetable consumption during meals may induce mechanical irritation from high-fibre vegetables on ulcer surfaces, thereby exacerbating psychological discomfort. This phenomenon may also relate to RAS population's positive therapeutic expectations regarding vegetables’ benefits for their condition. Such empirically driven dietary compliance, characterized by selective food intake based on experiential beliefs, frequently co-occurs with negative affect (53).

The findings of this study underscore the importance of personalized nutritional interventions, particularly for patients with oral chronic inflammatory diseases. during clinical practice, dietary modifications for RAS patients should comprehensively consider: (1) disease status (e.g., active RAS phases); (2) food composition and preparation methods; and (3) patients'subjective perceptions of food. Future prospective cohort studies are required to explore the relationships between dietary behaviours to psychological outcomes in RAS populations.

OHRQoL is utilized to quantify the impact of oral diseases on individuals’ overall health status, including functional, emotional, and social aspects (54). Oral health status is closely associated with OHRQoL (55). Our findings revealed that pain symptoms significantly impaired OHRQoL in RAS patients, indicating that RAS-related oral pain compromises their perception of well-being. Recurrent intraoral painful ulcers can lead to masticatory, swallowing, and speech dysfunction, thereby substantially compromising patients' quality of life (56). Our findings partially suggest that the potential anti-inflammatory properties of omega-3 polyunsaturated fatty acids may exert analgesic effects by reducing levels of inflammatory biomarkers [e.g., cytokines, eicosanoids, and C-reactive protein (CRP)], thereby significantly improving OHRQoL following RAS pain relief (57). High-frequency consumption of fried foods was associated with reduced OHRQoL in RAS patients, which aligns with prior research indicating that fast-food intake correlates with higher stress levels and stronger suicidal ideation (58). Patients with a demand for traditional medical interventions exhibited poorer OHRQoL. We hypothesize that this may result from dissatisfaction with the efficacy of prior conventional therapies, which could amplify psychological burden (59) and establish a negative feedback loop between treatment expectations and quality of life. For these unique populations attending TCM hospitals, it is essential to educate them about the intrinsic disease characteristics of RAS, to adjust treatment expectations, and to adopt a rational perspective toward the efficacy of traditional medical interventions. Moderate fruit intake is generally regarded as a component of a healthy diet, but this study observed an association with impaired OHRQoL. We hypothesize that this may relate to the local irritant effects of certain highly acidic fruits (e.g., citrus varieties). Acidic substances can reduce oral pH levels, disrupt mucosal barrier integrity, and exacerbate ulcer pain. These findings suggest the clinical necessity of personalized fruit selection recommendations (e.g., prioritizing low-acid options such as bananas or apples).

Given the methodological limitations of this study, the findings should be interpreted with caution. First, the relatively small sample size may affect statistical power and the reliability of conclusions. Second, the single-center design limited to a single national population substantially restricts the generalizability of the results. Furthermore, the reliance on self-reported measures introduces subjectivity as a non-negligible confounding factor when translating these findings into clinical practice-overestimation or underestimation of anxiety/depression levels in some cases may lead to biased assessments of disease severity. Thus, systematic evaluation of these confounding factors is essential when applying the results to clinical or research settings. Notably, despite these limitations, this study represents the first systematic exploration of multidimensional factors influencing anxiety, depressive symptoms, and OHRQoL in RAS patients, providing novel evidence for this field.

The findings emphasize that oral health practitioners should adopt a holistic medical perspective, viewing patients as integrated mind-body entities rather than focusing solely on localized oral treatments. When managing RAS patients, particular attention should be paid to assessing and addressing their psychological states to synergistically improve oral health outcomes and daily functional capacity. Future prospective longitudinal studies are required to validate these findings, thereby optimizing the clinical translation of research outcomes in this field. Notably, this study represents the first systematic exploration of multidimensional factors influencing anxiety, depressive symptoms, and OHRQoL in RAS population, providing a theoretical foundation for biopsychosocial integrated management models of the disease.

## 5 Conclusion

This study identifies factors such as ulcer characteristics, dietary habits, and the need of traditional medicine as influencing psychological distress and quality of life (QoL) in patients with RAS. Psychological distress serves as a significant trigger for recurrent ulcer episodes. Conversely, recurrent ulcers themselves can induce psychological distress and impair well-being, establishing psychological distress as a complication of RAS. Further questionnaire-based studies and clinical trials are warranted to inform the development of novel, personalized psychological interventions for RAS. Such interventions aim to improve the QoL and psychological well-being of RAS patients, thereby reducing ulcer recurrence frequency and improving clinical outcomes.

## Data Availability

The raw data supporting the conclusions of this article will be made available by the authors, without undue reservation.
